# A 12-year prospective study of stroke risk in older Medicare beneficiaries

**DOI:** 10.1186/1471-2318-9-17

**Published:** 2009-05-09

**Authors:** Fredric D Wolinsky, Suzanne E Bentler, Elizabeth A Cook, Elizabeth A Chrischilles, Li Liu, Kara B Wright, John F Geweke, Maksym Obrizan, Claire E Pavlik, Robert L Ohsfeldt, Michael P Jones, Robert B Wallace, Gary E Rosenthal

**Affiliations:** 1 Center for Research on the Implementation of Innovative Strategies into Practice, Iowa City Veterans Administration Medical Center, Iowa City, Iowa, USA; 2 Department of Health Management and Policy, College of Public Health, University of Iowa, Iowa City, Iowa, USA; 3 Department of Internal Medicine, Carver College of Medicine, University of Iowa, Iowa City, Iowa, USA; 4 Department of Epidemiology, College of Public Health, University of Iowa, Iowa City, Iowa, USA; 5 Department of Biostatistics, College of Public Health, University of Iowa, Iowa City, Iowa, USA; 6 Department of Economics, Tippie College of Business, University of Iowa, Iowa City, Iowa, USA; 7 Department of Geography, College of Liberal Arts and Sciences, University of Iowa, Iowa City, Iowa, USA; 8 Department of Health Management and Policy, School of Rural Public Health, Texas A&M University Health Science Center, College Station, Texas, USA

## Abstract

**Background:**

5.8 M living Americans have experienced a stroke at some time in their lives, 780K had either their first or a recurrent stroke this year, and 150K died from strokes this year. Stroke costs about $66B annually in the US, and also results in serious, long-term disability. Therefore, it is prudent to identify all possible risk factors and their effects so that appropriate intervention points may be targeted.

**Methods:**

Baseline (1993–1994) interview data from the nationally representative Survey on Assets and Health Dynamics among the Oldest Old (AHEAD) were linked to 1993–2005 Medicare claims. Participants were 5,511 self-respondents ≥ 70 years old. Two ICD9-CM case-identification approaches were used. Two approaches to stroke case-identification based on ICD9-CM codes were used, one emphasized sensitivity and the other emphasized specificity. Participants were censored at death or enrollment into managed Medicare. Baseline risk factors included sociodemographic, socioeconomic, place of residence, health behavior, disease history, and functional and cognitive status measures. A time-dependent marker reflecting post-baseline non-stroke hospitalizations was included to reflect health shocks, and sensitivity analyses were conducted to identify its peak effect. Competing risk, proportional hazards regression was used.

**Results:**

Post-baseline strokes occurred for 545 (9.9%; high sensitivity approach) and 374 (6.8%; high specificity approach) participants. The greatest static risks involved increased age, being widowed or never married, living in multi-story buildings, reporting a baseline history of diabetes, hypertension, or stroke, and reporting difficulty picking up a dime, refusing to answer the delayed word recall test, or having poor cognition. Risks were similar for both case-identification approaches and for recurrent and first-ever vs. only first-ever strokes. The time-dependent health shock (recent hospitalization) marker did not alter the static model effect estimates, but increased stroke risk by 200% or more.

**Conclusion:**

The effect of our health shock marker (a time-dependent recent hospitalization indicator) was large and did not mediate the effects of the traditional risk factors. This suggests an especially vulnerable post-hospital transition period from adverse effects associated with both their underlying health shock (the reasons for the recent hospital admission) and the consequences of their treatments.

## Background

In 2005, 2.6% of all living Americans (or 5.8 M) had experienced a stroke at some point in their lives [1]. Of these, 780K had experienced a first-ever or recurrent stroke that year, and 150K died from their strokes [1], maintaining stroke as the third leading cause of death in the US [2]. The substantial costs associated with stroke are projected to reach $66B in 2008, with a mean lifetime cost of about $140K per patient [1]. Stroke is also a leading cause of serious, long-term disability in the US [3], with survivors having significantly poorer physical and mental health component scores on the SF-12 [4], poorer EQ-5D scores [5], and poorer visual analog self-rated health scores [6] vs. controls [7].

*Healthy People 2010 *established national goals and objectives for reducing US annual stroke mortality rates by 20% from the 2000 baseline of 60 per 100K, as well as for promoting health behaviors that are known to reduce the risk of stroke itself, such as increasing awareness of stroke signs and symptoms, proper treatment and management for hypertension and diabetes, lowering and cholesterol levels, encouraging smoking cessation, and increasing physical activity levels [8]. Although progress toward the stroke mortality rate goal in the US has been excellent, reductions in the modifiable risk factors for stroke have varied and lag far behind the goals [9].

It is prudent, therefore, to identify all possible risk factors and to accurately calibrate their effects so that appropriate intervention points may be targeted [9]. Although the literature on stroke risk among older adults is extensive [1-7,10-20], few studies have simultaneously considered the roles of the sociodemographic, socioeconomic, place of residence, health behavior, disease history, and functional and cognitive status risk factors on stroke in large, nationally representative, prospective studies. We use data from the Survey on Assets and Health Dynamics among the Oldest Old (AHEAD) to do this.

Our study has six strengths. AHEAD is a large, nationally representative sample. Medicare claims (the public payor for health care among older adults in the US) were available for up to 12 years of post-baseline surveillance. Baseline AHEAD interviews (1993–1994) provided an extensive array of possible risk factors. The claims data allowed construction of a dynamic "health shock" measure reflecting the increased risk of stroke during various post-discharge transition periods following non-stroke hospitalizations. Two different approaches for stroke case-identification from the Medicare claims were considered and were further evaluated by replication after excluding participants with self-reported pre-baseline stroke histories. Finally, propensity score methods [21-24] were used to adjust for potential selection bias.

## Methods

### Data

Detailed descriptions of the AHEAD exist elsewhere [25-28]. Due to the multi-stage cluster and over-sampling of African Americans, Hispanics, and Floridians, all analyses were weighted to adjust for the unequal probabilities of selection. AHEAD included 7,447 age-eligible participants who were 70 years old or older at baseline, and had an 80.4% baseline response rate. Of these, 802 participants (10.8%) could not be linked to their Medicare claims, 604 participants (8.1%) were in managed Medicare at baseline, and 530 participants (7.1%) had proxy-respondents. This left 5,511 AHEAD self-respondents (74.0% of the total AHEAD cohort) in our analytic sample. Proxy-respondents were excluded because they did not complete the cognitive and psychosocial protocols which contain important possible risk factors for stroke. Participants in managed Medicare care plans were excluded because these plans do not have the same data-reporting requirements as fee-for-service Medicare plans [29]. That is, because they are reimbursed on a capitation basis, they do not have to submit claims for all services provided. Participants were censored at the time of two competing risks – death or enrollment into managed Medicare – whichever came first.

### Selection Bias

Propensity score methods were used to adjust for the potential selection bias introduced by these exclusions [21-24]. Simply put, a multivariable logistic regression model of inclusion in the analytic sample (included vs. not) was estimated among all 7,447 AHEAD participants. Predictors included all of the possible risk factors identified below, as well as others (a complete list is available on request). Model fit was good (C statistic = .72; Hosmer-Lemeshow statistic = .15 [30,31]). The average participation (P) rate (i.e., inclusion in the analytic sample) was determined within each propensity score (predicted probability) decile, and the inverse (1/P) was used to re-weight the data. This gives greater influence to participants in the analytic sample most like those not included. The propensity score weights were then rescaled so that the final weighted N equaled the number of participants in the analytic sample (i.e., 5,511; note that when the analyses were repeated using just the original AHEAD weights, the results were not meaningfully different, indicating that no selection bias occurred).

### Case Identification

Baseline interview dates were used to individually mark the beginning of the surveillance periods for each participant. Because there is considerable variation in approaches for using ICD9-CM diagnostic codes to identify stroke cases in administrative data [32], we used a slight modification (i.e., only primary admission codes) of two approaches suggested by Reker et al. [33], after which we imposed an additional constraint. The first case-identification approach has high (91%) sensitivity, but low (40%) specificity, while the second case-identification approach has low (54%) sensitivity but high (87%) specificity. The **high sensitivity **coding algorithm used any of the three following admission primary diagnoses for case-identification:

1) Primary diagnoses of subarachnoid hemorrhage, intracerebral hemorrhage, other intracranial hemorrhage, occlusion of cerebral arteries, acute but ill-defined cerebrovascular disease, or occlusion and stenosis of precerebral arteries.

2) Primary diagnosis of care involving use of rehabilitation procedures and a secondary diagnosis of one or more of the following – hemiplegia and hemiparesis; subarachnoid hemorrhage or intracerebral hemorrhage; other intracranial hemorrhage; occlusion and stenosis of precerebral arteries; occlusion of cerebral arteries; transient cerebral ischemia; acute but ill-defined, other and ill-defined, or late effects of cerebrovascular disease.

3) Primary diagnosis of occlusion and stenosis of precerebral arteries or transient cerebral ischemia and a secondary diagnosis of any of the following – hemiplegia and hemiparesis, subarachnoid hemorrhage, intracerebral hemorrhage, other intracranial hemorrhage, occlusion of cerebral arteries, or acute, but ill-defined cerebrovascular disease.

In contrast, the **high specificity **coding algorithm only used ICD9-CM codes for intracerebral hemorrhage, occlusion and stenosis of precerebral arteries, and occlusion of cerebral arteries for case-identification.

We then placed an additional constraint on both approaches. First, we estimated the high sensitivity and high specificity approaches using all 5,511 participants and included a binary marker reflecting any self-reported history of pre-baseline stroke. This allowed us to focus solely on post-baseline (i.e., new) strokes that were either first-ever or recurrent strokes. Second, we re-estimated both models after excluding participants with self-reported pre-baseline stroke history. This allowed us to focus solely only on post-baseline first-ever strokes.

Strokes had to occur at least one day after the participant's baseline interview, and censoring occurred at death or enrollment into managed Medicare using data from the Medicare claims denominator file, because at these points the participant's observation period ended. Multivariable proportional hazards regression with competing risks [34] was used to model time to stroke, assuming that these competing risks were independent and censored. Model development and evaluation followed standard procedures [35,36].

### Baseline Risk Factors

Established risks for stroke include both modifiable and non-modifiable factors [1-7,10-20]. Modifiable risk factors (i.e., those that the American Heart Association [1] identifies as able to be "changed, treated, or controlled") include hypertension, heart disease, diabetes, smoking, dyslipidemia, obesity, physical inactivity, and alcohol abuse. Non-modifiable factors include increased age, sex, race, prior strokes and TIAs, family history, asymptomatic carotid bruit, geography, and socioeconomic status. These risk factors can be categorized into sociodemographic, socioeconomic, place of residence, health behavior, disease history, and functional and cognitive status. To broaden the potential net, we included additional (possible) risk factors in each category when these were available in the AHEAD at baseline.

Sociodemographic factors included age, sex, race, living alone, marital status, and the importance of religion. Socioeconomic factors included education, income, total wealth, the number of health insurance policies, and perceived neighborhood safety. Place of residence was measured by population density, geographic region, and dwelling type. Health behaviors included the body mass index (BMI), smoking history, and current alcohol consumption.

Disease history included whether the participant had ever been told by a physician that s/he had angina, arthritis, cancer, diabetes, a heart attack, heart disease (including coronary heart disease, angina, congestive heart failure, or other heart problems), a previous hip fracture, hypertension or high blood pressure, lung disease, psychological problems, or a stroke or TIA, as well as a binary comorbidity marker reflecting having four or more of these conditions. We also included whether the participant was hospitalized in the year prior to baseline, and the number of physician visits during that period as indirect indicators of otherwise unmeasured disease burden. Functional status was measured by self-rated health, counts of the number of difficulties with activities of daily living (ADLs) and instrumental ADLs (IADLs), the number of reported depressive symptoms, falling in the year prior to baseline, reports of bothersome pain, the ability to pick up a dime, and self-rated vision, memory, and urinary incontinence. Cognitive status included immediate and delayed word recalls, and the Telephone Interview to Assess Cognitive Status [TICS; [37]].

Although extensive, our list of baseline risk factors (both traditional and novel) is incomplete. We lack measures of family history, dyslipidemia, and asymptomatic carotid bruit. Although these are known contributors to the prediction of stroke risk among older adults, we are unaware of any evidence to suggest that their absence appreciably alters the risk estimates obtained for other covariates in multivariable models. Thus, their omission is unlikely to have biased our parameter estimates.

### Health Shocks

We introduced a dynamic element to the analysis by constructing a "health shock" marker [38] using the post-baseline Medicare claims for each participant. This time-dependent covariate was switched "on" at any time prior to censoring when the participant was admitted to a hospital for any primary ICD9-CM diagnosis other than stroke. It stayed "on" for n days after discharge, and was then switched "off." It could subsequently be switched back "on" at the onset of another pre-censoring hospital admission for something other than stroke. Because two separate case-identification approaches were used, we used health shock markers that were approach-specific. These separate measures (depending on the case-identification approach) capture the transition period when older adults are especially vulnerable to adverse effects associated with both their underlying health shock (i.e., the reasons for their non-stroke admission) and the consequences of their treatments, especially in fragmented health care delivery systems like the US [39-44]. Sensitivity analyses were conducted to determine which of several values of n was most predictive of stroke – 7, 14, 30, 60, 90, and 180 days and 1, 1.5 and 2 years.

### Institutional Review Board (IRB) Approval

The research reported here is supported by an NIH grant (R01 AG022913) to Dr. Wolinsky. The research and restricted data protection plans associated with this grant were approved by AHEAD on February 20, 2003 (#2003-006). Furthermore, the human subject protocol for this US National Institutes of Health grant was fully approved by University of Iowa IRB-01 on March 24, 2003, and was fully approved again by IRB-01 at each of its annual reviews. A Data Use Agreement with the US Centers for Medicare and Medicaid Services (CMS; DUA 14807) for this study was fully approved on March 3, 2005.

## Results

### Descriptive

Tables 1, 2, 3 contain the overall percentages (or means) for each of the traditional and novel stroke risk factors that were considered, as well as the percentages (or means) among participants without strokes, and with strokes based on the high sensitivity and high specificity case-identification strategy. In the overall analytic sample, the mean age was 77, 38% were men, 10% were African American, 4% were Hispanic, and 41% were widowed. One-fourth had only been to grade school, and mean income was $25K. One-fourth had arthritis, 9% had angina, 13% had cancer, 12% had diabetes, and 46% had hypertension. Pre-baseline stroke histories were reported by 472 participants (8.6%). Post-baseline health shocks were experienced by 81%, and post-baseline strokes were experienced by 9.9% (N = 545) and 6.8% (N = 374), depending on whether the high sensitivity or high specificity approach was used for case-identification. Of these post-baseline strokes, 456 and 323 first-ever strokes were identified using the high sensitivity approach and the high specificity approach, respectively. The total number of person-years of surveillance was 38,992 with a mean of 7.1 per-person. To conserve space and because multivariable hazards models (see below) will be used to identify the adjusted or net risks associated with each factor, we do not discuss crude differences across stroke vs. non-stroke categories here (i.e., columnar differences within rows).

**Table 1:** Percentages or means for each of the baseline sociodemographic and socioeconomic stroke risk factors overall and by stroke classification.

**Variable**	**All Subjects** **N = 5511**	**Non-Stroke Subjects** **N = 4966**	**Stroke Sensitivity Cases** **N = 545**	**Stroke Specificity Cases** **N = 374**
***Sociodemographics***				
Age in years				
69 – 74 (RG)	38%	39%	34%	37%
75 – 79	29%	29%	31%	28%
80 – 84	19%	19%	25%	25%
85 +	13%	13%	11%	10%
Sex (men)	38%	38%	35%	36%
Race				
African American	10%	10%	13%	13%
Hispanic	4%	4%	5%	6%
White (RG)	85%	85%	82%	81%
Lives Alone	37%	36%	39%	37%
Marital Status				
Widowed	41%	41%	47%	45%
Divorced/Separated	5%	5%	4%	4%
Never Married	3%	3%	5%	6%
Married (RG)	50%	51%	43%	46%
Religion Not Important	11%	11%	13%	13%
***Socioeconomics***				
Education				
Grade School	25%	25%	28%	25%
High School (RG)	48%	48%	49%	49%
College	27%	27%	23%	26%
Income				
Quintile 1 (≤ $7500)	16%	15%	20%	19%
Quintile 2 ($7501 – $14,999)	30%	30%	30%	29%
Quintile 3 ($15,000 – $19,500)	13%	13%	13%	12%
Quintile 4 ($19,501 – $30,000)	19%	19%	18%	17%
Quintile 5 (> $30,000)	23%	23%	20%	23%
Total Wealth	$174,542	$176,062	$160,706	$186,779
# of Health Insurance Policies	0.86	0.87	0.83	0.84
Neighborhood Safety				
Poor	3%	3%	3%	3%
Fair	9%	9%	11%	11%
Good to Excellent (RG)	87%	87%	86%	86%

Notes for Tables 1–3: Among 5,511 AHEAD self-respondents (at baseline) with linked Medicare claims who were not in managed care at their baseline interviews. All variables were binary coded (1 = yes, 0 = no) except total wealth (actual dollar value) and the number of health insurance policies (actual number). RG = Reference Group.

**Table 2:** Percentages or means for each of the baseline residence, health behavior, and disease stroke risk factors overall and by stroke classification.

**Risk Factors**	**All Subjects** **N = 5511**	**Non-Stroke Subjects** **N = 4966**	**Stroke Sensitivity Cases** **N = 545**	**Stroke Specificity Cases** **N = 374**
***Residence Characteristics***				
Population over 1,000,000	47%	47%	46%	51%
Region of the US				
Northeast	22%	23%	21%	24%
North Central	27%	26%	29%	28%
West	18%	19%	14%	13%
South (RG)	33%	32%	36%	35%
Type of Residence				
Multiple Story Home	39%	39%	45%	47%
Mobile Home	6%	7%	6%	5%
Single Story Non-mobile (RG)	53%	54%	49%	48%
***Health Behaviors***				
Body Mass				
Obese	14%	13%	18%	20%
Overweight	36%	37%	34%	35%
Normal (RG)	46%	46%	44%	43%
Underweight	4%	4%	4%	2%
Smoking History				
Former Smoker	42%	42%	40%	40%
Current Smoker	10%	10%	8%	7%
Never Smoked (RG)	48%	48%	52%	53%
Drinking History				
3 or more drinks/day	2%	2%	1%	2%
1 – 2 drinks/day	9%	9%	8%	9%
Less than 1 drink/day (RG)	89%	89%	91%	89%
***Disease History***				
Angina	9%	9%	10%	10%
Arthritis	25%	25%	25%	24%
Cancer	13%	13%	11%	13%
Diabetes	12%	12%	19%	21%
Heart Attack	7%	7%	8%	8%
Heart Disease	29%	28%	34%	32%
Hip Fracture	5%	5%	5%	5%
Hypertension	46%	45%	53%	55%
Lung Disease	9%	10%	8%	7%
Psych. Problems	7%	7%	7%	6%
Stroke	10%	9%	16%	14%
4 or more of the above conditions	12%	11%	15%	15%
Hospitalized in past year	23%	22%	26%	24%
Visited a Doctor in the past year				
0 – 1 time (RG)	23%	23%	20%	20%
2 – 3 times	28%	28%	26%	28%
4 – 5 times	20%	20%	21%	18%
6 or more times	28%	28%	33%	34%

**Table 3:** Percentages or means for each of the baseline functional and cognitive status stroke risk factors overall and by stroke classification.

**Risk Factors**	**All Subjects** **N = 5511**	**Non-Stroke Subjects** **N = 4966**	**Stroke Sensitivity Cases** **N = 545**	**Stroke Specificity Cases** **N = 374**
***Functional Status***				
Self-Rated Health Status				
Poor vs. Fair to Excellent	12%	12%	16%	14%
ADL Count				
0 (RG)	81%	81%	76%	79%
1	10%	10%	13%	13%
2	4%	4%	6%	5%
3 or more	4%	4%	5%	3%
IADL Count				
0 (RG)	79%	79%	75%	78%
1	11%	11%	10%	11%
2	5%	5%	7%	6%
3 or more	6%	5%	8%	5%
CESD-8 Count				
0 (RG)	38%	38%	36%	38%
1	22%	22%	22%	23%
2	14%	14%	11%	11%
3 or more	26%	26%	31%	28%
Any Falls Past Year	25%	25%	27%	22%
Bothersome Pain	33%	32%	35%	33%
Difficulty Picking Up a Dime	8%	8%	13%	12%
Vision (self-rated)				
Poor	9%	8%	10%	8%
Fair	17%	17%	18%	17%
Good to Excellent (RG)	74%	75%	72%	75%
Memory (self-rated)				
Poor	6%	6%	8%	6%
Fair	20%	20%	24%	23%
Good to Excellent (RG)	74%	74%	68%	71%
Urinary Incontinence	19%	19%	18%	18%
***Cognitive Status***				
Low 1/2 Imm. Word Rec.	49%	49%	55%	56%
High 1/2 Imm. Word Rec.	48%	48%	40%	41%
Refused to Answer Imm. Rec.	3%	3%	5%	3%
Low 1/2 Del. Word Rec.	55%	54%	59%	60%
High 1/2 Del. Word Rec.	41%	43%	35%	35%
Refused to Answer Del. Rec.	4%	3%	6%	5%
TICS-7 Score				
Good Cognition (vs. Poor)	79%	80%	73%	75%

### Sensitivity Analyses of the Health Shock Indicator

Because our use of the health shock indicator is novel [38], we conducted extensive sensitivity analyses using the health shock measure to predict stroke, adjusting for the possible risk factors described above. Our purpose was to determine which of several values of n was most predictive of stroke – 7, 14, 30, 60, 90, and 180 days and 1, 1.5 and 2 years. The results (all p values < .001) of these analyses are shown in Table 4, separately for the high sensitivity and high specificity case-identification approaches, and with and without including participants who had self-reported histories of pre-baseline strokes.

**Table 4:** Adjusted^a ^hazard ratios for the health shock indicator using different post-discharge time periods.

	**Stroke Definition: High Sensitivity**		**Stroke Definition: High Specificity**	
**Time after Discharge from Hospital**	**All**	**No Prior Report of Stroke**	**All**	**No Prior Report of Stroke**
7 days	5.36	5.80	2.90	3.37
14 days	4.65	4.98	3.10	3.41
30 days	3.53	3.60	2.82	2.94
60 days	3.04	3.23	2.73	2.95
90 days	2.98	3.13	2.78	2.98
6 months	2.75	2.89	2.49	2.66
1 year	2.37	2.50	2.09	2.19
1.5 years	2.16	2.18	1.89	1.87
2 years	2.01	2.05	1.78	1.79

^a ^All Hazard Ratios are adjusted by the covariates significant in the final models for each group.

^b ^All Adjusted Hazard Ratios presented in this table are statistically significant at the p < .001 level.

As shown, when using the high specificity case-identification approach (columns 3 and 4), the results (AHRs for the health shock indicator) are relatively robust (i.e., of consistent magnitude) reflecting a trebling of the risk for stroke regardless of whether the time-dependent measure was allowed to stay "switched on" for only 7 days after discharge from the prior hospitalization for something other than stroke, out to at least six months afterwards. Moreover, this holds regardless of whether recurrent and first-ever (column 3), or just first-ever strokes (column 4) were considered.

In contrast, when using the high sensitivity case-identification approach (columns 1 and 2), the results are only robust in the sense that at least a doubling of the risk for stroke was observed. While the magnitude of that risk was greatest when the time-dependent measure was allowed to stay "switched on" for only 7 days after discharge from the prior hospitalization (AHRs = 5.36 and 5.80 in columns 1 and 2), that risk declined by about half within 90 days afterwards. This lack of robustness likely reflects the higher false positive rate associated with the high sensitivity case-identification approach. Based on these findings, we present below the results from analyses using both the 90 and 7 day calibrations for how long after the prior hospital discharge for something other than stroke the time-dependent health shock marker was allowed to remain "switched on."

### Multivariable Hazards Models

Additional file 1 contains the results from four models using the high sensitivity case-identification approach. Column 1 contains the crude hazards ratios (AHRs), column 2 contains the adjusted HRs (AHRs) from a static trimmed model, and columns 3 and 4 contain the AHRs from the static trimmed model (column 2) after introducing the dynamic health shock marker calibrated separately at 90 and 7 days, respectively. These HRs and AHRs were estimated among all 5,511 participants in the analytic sample and included only those baseline risk factors independently significant at the .05 level or beyond (consolidated from forward, backward, and stepwise modeling). Columns 5, 6, 7, and 8 contain the results when this analysis was replicated after excluding the 456 participants who had self-reported pre-baseline stroke histories. Because there was no meaningful difference in the risk estimates for the static factors depending on whether the 90- or 7-day calibration was used for the health shock marker, and because the largest effect for the health shock marker was observed for the 7-day calibration, we only discuss the effects of the 7-day calibration here.

The results for the **high sensitivity case-identification **static model (column 2) indicate that the risks of post-baseline stroke (either recurrent or first-ever) were statistically significantly elevated for 80–84 year olds (vs. 69–74 year olds; AHR = 1.63), widowed or never married participants (vs. married; AHRs = 1.22 and 1.59), individuals for whom religion was not important (AHR = 1.36), participants living in multi-story buildings (vs. single story non-mobile homes; AHR = 1.39), individuals reporting a baseline history of diabetes (AHR = 1.71), heart disease (AHR = 1.37), hypertension (AHR = 1.20), or stroke (AHR = 2.01), participants reporting poor or fair health (AHRs = 1.42 and 1.26), individuals reporting difficulty picking up a dime (AHR = 1.42), and participants who refused to answer the delayed word recall test (vs. those in the upper half; AHR = 1.96). At the same time the risks of post-baseline stroke (either recurrent or first-ever) were statistically significantly reduced for participants with good cognition scores (AHR = 0.80). Introduction of the 7-day calibration of the dynamic health shock (recent hospitalization) marker (column 4) did not appreciably alter any of these risk estimates, although it did significantly improve the fit of the model (p < .001 for the chi-square improvement in the log likelihood ratio), as reflected in its statistically significant AHR of 5.36. When these analyses were repeated (columns 5, 6, 7, and 8) after excluding those reporting pre-baseline stroke histories (i.e., focusing only on first-ever strokes), the results were essentially the same.

Additional file 2 contains the results obtained when the high **specificity case-identification **approach was used. Again, because there was no meaningful difference in the risk estimates for the static factors depending on whether the 90- or 7-day calibrations was used for the health shock marker, we only discuss the effects of the short calibration here. The results in the static model (column 2) indicate that the risks of post-baseline stroke (either recurrent or first-ever) were statistically significantly elevated for 80–84 year olds (vs. 69–74 year olds; AHR = 1.68), never married participants (vs. married; AHR = 1.70), participants living in multi-story buildings (vs. single story non-mobile homes; AHR = 1.42), individuals reporting a baseline history of diabetes (AHR = 2.01), hypertension (AHR = 1.38), or stroke (AHR = 1.81), and participants reporting difficulty picking up a dime (AHR = 1.45). At the same time the risks of post-baseline stroke (either recurrent or first-ever) were statistically significantly reduced for participants having only a grade school education (AHR = 0.68), and for individuals having good cognition scores (AHR = 0.74). Introduction of the 7-day calibration of the dynamic health shock (recent hospitalization) marker (column 4) did not appreciably alter any of these risk estimates, although it did significantly improve the fit of the model (p < .001 for the chi-square improvement in the log likelihood ratio), as reflected in its statistically significant AHR of 2.90. When these analyses were repeated (columns 5, 6, 7, and 8) after excluding those reporting pre-baseline stroke histories (i.e., focusing on first-ever strokes only), the results were essentially the same, with the exception of the identification of the high risk associated with obesity (vs. normal weight; AHR = 1.53).

## Discussion

Four points warrant further discussion. First, our study has a number of strengths. AHEAD is a nationally representative sample of 7,447 individuals who were 70 years old or older when they completed their baseline interviews in 1993–1994. We linked an extensive array of possible risk factors (both traditional and novel) obtained from the baseline interview data for 5,511 of the AHEAD participants to their Medicare claims for up to 12 years of post-baseline surveillance, and we used propensity score methods to adjust for potential selection bias in the analytic sample. From the claims data we constructed a time-dependent (dynamic) post-baseline "health shock" (recent hospitalization) measure and included it in our multivariable hazards models. Two different approaches for stroke case-identification were considered, one that emphasized sensitivity and one that emphasized specificity, and with both approaches we replicated our analyses after excluding participants who self-reported pre-baseline stroke histories. Among the 5,511 participants, 6.8% and 9.9% suffered a post-baseline stroke (recurrent or first-ever), depending on whether the high specificity or high sensitivity (respectively) case-identification approach was used.

The second point that warrants discussion involves the importance of the dynamic health shock marker [38], which was measured indirectly using the time-dependent recent hospitalization indicator. This effect was quite large and did not mediate the effects of the baseline stroke risk factors. It captures the transition period when older adults are especially vulnerable to adverse effects associated with both their underlying health shock (i.e., the reasons for their recent hospital admission) and the consequences of their treatments, especially in fragmented health care delivery systems [39-43]. When calibrated at 7 days, which is when the effect size peaked, the health shock measure increased the risk of stroke by about 200–480% (depending on the case-identification approach and restriction to first-ever strokes), and substantially improved model fit. This suggests that post-discharge planning and monitoring for a week or so following hospital discharge for something other than a stroke might be fruitful and might potentially reduce the risk of subsequent stroke during this transition period. It would seem prudent at this point to design and evaluate a pilot, short-term, post-discharge planning and monitoring intervention study, consistent with the recent work and suggestions of Coleman and colleagues [45-47].

Although the introduction of the health shock (i.e., recent hospitalization) measure is a very promising development that underscores the need to shift from static to dynamic risk modeling approaches [38], further research is needed. That research should explore the health shock measure in order to clarify what the underlying etiologic mechanism(s) might be. Such research should include whether restrictions to surgical vs. medical admissions, shorter vs. longer stays, or other decompositions would identify particular hospitalization subsets that pose the greatest risks for subsequent stroke.

The third discussion point involves the identification of what we did find; that is, the static baseline risk factors and the magnitudes of their risks. Regardless of the case-identification approach used (high sensitivity vs. high specificity) and whether first-ever **and **recurrent strokes or just first-ever strokes were considered, our risk estimates were remarkably consistent. The greatest risks involved increased age, individuals who were widowed or never married, participants living in multi-story buildings, individuals reporting a baseline history of diabetes, hypertension, or stroke, and participants who reported difficulty picking up a dime, refused to answer the delayed word recall test, or who had poor cognition scores. With two exceptions, what we found is generally consistent with the extant literature [1-7,10-20].

The two exceptions involve the risks associated with multi-dwelling residence and the protective effect of angina. The significant stroke risk associated with multi-story residential dwellings vs. single-story residential dwellings has not previously been reported in the literature. This 40% increased risk was also remarkably consistent regardless of the case-identification approach and the type of strokes considered. Because the point estimates (AHRs) obtained for the other dwelling unit contrasts approximate unity (vs. AHRs ≥ 1.39 for this specific dwelling unit contrast), these differences are not due to insufficient statistical power. Furthermore, because the same differential dwelling unit contrast pattern was observed among the crude HRs, these results are not due to statistical over-adjustment or to multicollinearity. We believe that the increased risk of multi-story residential dwelling reflects the greater physical, social, and psychological burdens faced by older adults in those settings. Although this interpretation is consistent with the literature on congested living and stress [48-50], replication of these results using other nationally representative samples and similar residential dwelling contrasts will be necessary to move this interpretation beyond *post hoc *speculation.

The protective effect observed for angina is less straightforward for two reasons. First, the independent effect is only manifest with the high sensitivity definition when both those with first-ever and recurrent strokes are considered. Second, the statistically significant independent effect of angina is protective, while it's statistically significant crude effect placed participants at risk. Based on these facts, we assume that the observed protective effect of angina is a statistical artifact, possibly due to statistical over-adjustment.

Our fourth discussion point involves what we did not find. Specifically, we did not observe elevated risks for men, minorities, geographic region, socioeconomic gradients, or obese participants, despite the fact that these are generally reported in the literature [1-7,10-20]. Determining why elevated risks for these factors were not observed in the AHEAD is beyond the scope of the present study. Nonetheless, we are convinced that this is not an artifact of statistical over-adjustment because these factors exhibited no crude associations with post-baseline strokes. We also expect that given our sample size and the distributions of these risk factors in the AHEAD, these non-findings due not result from insufficient statistical power. Further research, however, using other nationally representative samples will be necessary to resolve these issues.

Finally, in concluding this article, we note the three major limitations of our study. First, family history, dyslipidemia, and asymptomatic carotid bruit were not available for inclusion in the analysis. Second, although the AHEAD is rich in self-reported data and linked to Medicare claims for more than a decade, detailed clinical histories were not available, restricting our study to an epidemiologic vs. etiologic analysis. Lastly, we relied solely on baseline (i.e., static) risk factors from the AHEAD self-reports, even though several of them (such as ADLs, IADLs, and self-rated health) were repeated at most follow-ups. Inclusion of those repeated self-reports, however, would have created numerous additional complexities involving missing data, selection bias, and correlated error structures.

## Conclusion

The effect of our dynamic health shock marker (a time-dependent recent hospitalization indicator) was large and did not mediate the effects of the traditional risk factors. This suggests an especially vulnerable post-hospital transition period from adverse effects associated with both their underlying health shock (the reasons for the recent hospital admission) and the consequences of their treatments. Based on these results, designing and piloting a short-term, post-discharge planning and monitoring intervention consistent with the recent work and suggestions of Coleman and colleagues [48-50] may be warranted.

## Competing interests

The authors declare that they have no competing interests.

## Authors' contributions

FW conceived of the study, wrote the grant application, designed the analyses, interpreted the results, and revised the manuscript. SB and EC conducted all analyses at FW's direction. MJ, JG, and RO assisted in the design and oversight of the statistical analyses and their interpretation. LL assisted with all data linkage, merging, and recoding. MO conducted the propensity score modeling under the direction of JG. EC participated in the original design of the study, and with KW assisted in the oversight of the data merging and recoding components for the administrative claims data. CP harvested and prepared all of the geocoded information from Census and other sources for migration and linkage. GR and RW participated in the conceptualization of the grant application and the overall study design, and provided clinical expertise throughout the study. All authors participated in numerous meetings to outline, read, critique, revise, re-read, and grant approval the final manuscript. FW and SB had full access to all of the data in the study and take responsibility for the integrity of the data and the accuracy of the data analysis. All authors read and approved the final manuscript.

## Pre-publication history

The pre-publication history for this paper can be accessed here:



## Supplementary Material

Additional file 1 **Supplemental table 1**. Crude hazards ratios (HR), adjusted static HRs (AHRs), and adjusted dynamic AHRs obtained using the high sensitivity^a ^case-identification approach. Click here for file

Additional file 2 **Supplemental table 2**. Crude hazards ratios (HR), adjusted static HRs (AHRs), and adjusted dynamic AHRs obtained using the high specificity^a ^case-identification approach. Click here for file
