# Supplementary material for: A 12-year prospective study of stroke risk in older Medicare beneficiaries
Source: BMC Geriatr. 2009 May 9;9:17. doi: 10.1186/1471-2318-9-17 (PMC2683849; doi:10.1186/1471-2318-9-17)
Supplement: Additional file 1 — Supplemental table 1. Crude hazards ratios (HR), adjusted static HRs (AHRs), and adjusted dynamic AHRs obtained using the high sensitivitya case-identification approach. [file 1471-2318-9-17-S1.doc]

Table 5. Crude hazards ratios (HR), adjusted static HRs (AHRs), and adjusted dynamic AHRs 0btained using the high sensitivity^a^ case-identification approach.

|  | **All Persons**  **(N=5511)^b^** | | | | **Persons Without Baseline Self-Reported Stroke (N=4987)^c^** | | | |
| --- | --- | --- | --- | --- | --- | --- | --- | --- |
| **Risk Factors** | **Crude HRs** | **Static AHRs** | **Dynamic AHRs** | **Dynamic**  **AHRs** | **Crude HRs** | **Static AHRs** | **Dynamic AHRs** | **Dynamic AHRs** |
| ***Sociodemographics*** |  |  |  |  |  |  |  |  |
| Age in years  69 – 74 (Ref)  75 – 79  80 – 84  85 + | 1.00  1.35 **  1.97 ***  1.56 ** | 1.00  1.21  1.63 ***  1.16 | 1.00  1.20  1.60 ***  1.11 | 1.00  1.21  1.61 ***  1.13 | 1.00  1.27 *  2.00 ***  1.51 * | 1.00  1.18  1.77 ***  1.19 | 1.00  1.16  1.71 ***  1.13 | 1.00  1.17  1.74 ***  1.15 |
| Marital Status  Widowed  Divorced/Separated  Never Married  Married (ref) | 1.46 ***  1.04  1.96 ***  1.00 | 1.22 *  0.87  1.59 *  1.00 | 1.22 *  0.87  1.58 *  1.00 | 1.22 *  0.87  1.57 *  1.00 | 1.45 ***  1.05  2.22 ***  1.00 | 1.23 *  0.91  1.78 **  1.00 | 1.22  0.92  1.76 **  1.00 | 1.22  0.91  1.75 *  1.00 |
| Religion Not Important | 1.23 | 1.36 * | 1.38 * | 1.37 * | NA | NA | NA | NA |
| ***Residence Characteristics*** |  |  |  |  |  |  |  |  |
| Region of the US  Northeast  North Central  West  South (REF) | 0.94  0.94  0.77  1.00 | 0.81  0.92  0.84  1.00 | 0.80  0.92  0.84  1.00 | 0.79  0.92  0.84  1.00 | NA | NA | NA | NA |
| Type of Residence  Multiple Story Dwelling  Mobile Home  Single Story Non-mobile (REF) | 1.32 **  1.16  1.00 | 1.39 ***  1.10  1.00 | 1.39 ***  1.07  1.00 | 1.39 ***  1.08  1.00 | 1.40 ***  1.33  1.00 | 1.38 **  1.33  1.00 | 1.38 **  1.31  1.00 | 1.38 **  1.32  1.00 |

Table 5. Continued.

|  | **All Persons**  **(N=5511)^b^** | | | | **Persons Without Baseline Self-Reported Stroke (N=4987)^c^** | | | |
| --- | --- | --- | --- | --- | --- | --- | --- | --- |
| **Risk Factors** | **Crude HRs** | **Static AHRs** | **Dynamic AHRs** | **Dynamic**  **AHRs** | **Crude HRs** | **Static AHRs** | **Dynamic AHRs** | **Dynamic AHRs** |
| ***Health Behaviors*** |  |  |  |  |  |  |  |  |
| Body Mass  Obese  Overweight  Normal (ref)  Underweight | 1.30 *  0.88  1.00  1.22 | 1.12  0.85  1.00  0.93 | 1.12  0.85  1.00  0.93 | 1.12  0.85  1.00  0.93 | 1.38 *  0.89  1.00  0.96 | 1.29  0.89  1.00  0.83 | 1.27  0.89  1.00  0.82 | 1.27  0.89  1.00  0.83 |
| ***Disease History*** |  |  |  |  |  |  |  |  |
| Angina | 1.39 * | 0.74 | 0.72 * | 0.73 | 1.28 | 0.73 | 0.71 | 0.71 |
| Arthritis | NA | NA | NA | NA | 1.00 | 0.74 * | 0.74 * | 0.74 * |
| Diabetes | 2.05 *** | 1.71 *** | 1.65 *** | 1.68 *** | 2.08 *** | 1.68 *** | 1.60 *** | 1.63 *** |
| Heart Disease | 1.60 *** | 1.37 ** | 1.30 * | 1.34 ** | 1.61 *** | 1.50 *** | 1.42 ** | 1.47 *** |
| Hypertension | 1.43 *** | 1.20 * | 1.18 | 1.19 | 1.38 *** | 1.25 * | 1.23 * | 1.24 * |
| Lung Disease | NA | NA | NA | NA | 0.86 | 0.74 | 0.70 | 0.72 |
| Stroke | 2.65 *** | 2.01 *** | 2.00 *** | 2.01*** | NA | NA | NA | NA |
| Health Shock (7 day) | 6.50 *** | -- | -- | 5.36 *** | 6.93 *** | -- | -- | 5.80 *** |
| Health Shock (90 day) | 3.52 *** | -- | 2.98 *** | -- | 3.62 *** | -- | 3.13 *** | -- |
| ***Functional Status*** |  |  |  |  |  |  |  |  |
| Self-Rated Health Status  Poor  Fair  G/VG/E (REF) | 2.51 ***  1.69 ***  1.00 | 1.42 *  1.26 *  1.00 | 1.33  1.23  1.00 | 1.37 *  1.24 *  1.00 | 2.25 ***  1.64 ***  1.00 | 1.65 **  1.39 **  1.00 | 1.51 *  1.35 **  1.00 | 1.56 **  1.36 **  1.00 |
| ADL Count  0 (REF)  1  2  3 or more | 1.00  1.74 ***  2.02 ***  1.78 ** | 1.00  1.25  1.12  0.82 | 1.00  1.23  1.07  0.80 | 1.00  1.24  1.08  0.81 | NA | NA | NA | NA |
| Diff. Picking up a Dime | 1.96 *** | 1.42 * | 1.43 * | 1.42 * | 1.67 ** | 1.53 * | 1.52 * | 1.52 * |

Table 5. Continued.

|  | **All Persons**  **(N=5511)^b^** | | | | **Persons Without Baseline Self-Reported Stroke (N=4987)^c^** | | | |
| --- | --- | --- | --- | --- | --- | --- | --- | --- |
| **Risk Factors** | **Crude HRs** | **Static AHRs** | **Dynamic AHRs** | **Dynamic**  **AHRs** | **Crude HRs** | **Static AHRs** | **Dynamic AHRs** | **Dynamic AHRs** |
| Incontinence | NA | NA | NA | NA | 0.92 | 0.79 | 0.78 | 0.78 |
| ***Cognitive Status*** |  |  |  |  |  |  |  |  |
| Low ½ Imm. Word Rec.  Refused to Answer Imm. Rec. | 1.62 ***  2.43 *** | 1.16  0.96 | 1.15  0.97 | 1.16  0.97 | NA | NA | NA | NA |
| Low ½ Del. Word Rec.  Refused to Answer Del. Rec. | 1.60 ***  2.76 *** | 1.13  1.96 * | 1.12  1.90 * | 1.12  1.93 * | 1.48 ***  2.62 *** | 1.16  1.99 ** | 1.15  1.92 ** | 1.15  1.96 ** |
| TICS-7 Score  Good Cognition | 0.56 *** | 0.80 * | 0.80 * | 0.80 * | 0.59 *** | 0.78 * | 0.79 * | 0.79 * |

*p<.05; **p<.01; ***p<.001

^a^High Sensitivity Algorithm:

1. Primary diagnoses of subarachnoid hemorrhage, intracerebral hemorrhage, other intracranial hemorrhage, occlusion of cerebral arteries, acute but ill-defined cerebrovascular disease, or occlusion and stenosis of precerebral arteries OR
2. Primary diagnosis of care involving use of rehabilitation procedures and a secondary diagnosis of one or more of the following—hemiplegia and hemiparesis; subarachnoid hemorrhage, intracerebral hemorrhage; other intracranial hemorrhage; occlusion and stenosis of precerebral arteries; occlusion of cerebral arteries; transient cerebral ischemia; acute but ill-defined, other and ill-defined, or late effects of cerebrovascular disease OR
3. Primary diagnosis of occlusion and stenosis of precerebral arteries or transient cerebral ischemia and a secondary diagnosis of any of the following— hemiplegia and hemiparesis, subarachnoid hemorrhage, intracerebral hemorrhage, other intracranial hemorrhage, occlusion of cerebral arteries, or acute, but ill-defined cerebrovascular disease.

^b^Among these 5,511 AHEAD self-respondents (at baseline), 545 (9.9%) experienced one or more strokes post-baseline and prior to any managed care enrollment.

^c^Among these 4,987 AHEAD self-respondents (at baseline) who did NOT self-report pre-baseline strokes, 456 (9.1%) experienced one or more strokes post-baseline and prior to any managed care enrollment.

Note: Ref = reference group, NA = not applicable (i.e., risk factor did not make it into this final model).
