# Supplementary material for: A 12-year prospective study of stroke risk in older Medicare beneficiaries
Source: BMC Geriatr. 2009 May 9;9:17. doi: 10.1186/1471-2318-9-17 (PMC2683849; doi:10.1186/1471-2318-9-17)
Supplement: Additional file 2 — Supplemental table 2. Crude hazards ratios (HR), adjusted static HRs (AHRs), and adjusted dynamic AHRs obtained using the high specificitya case-identification approach. [file 1471-2318-9-17-S2.doc]

Table 6. Crude hazards ratios (HR), adjusted static HRs (AHRs), and adjusted dynamic AHRs obtained using the high specificity^a^ case-identification approach.

|  | **All Persons**  **(N=5,511)^b^** | | | | **Persons Without Baseline Self-Reported Stroke (N=4,987)^c^** | | | |
| --- | --- | --- | --- | --- | --- | --- | --- | --- |
| **Risk Factors** | **Crude HRs** | **Static AHRs** | **Dynamic AHRs** | **Dynamic AHRs** | **Crude HRs** | **Static AHRs** | **Dynamic AHRs** | **Dynamic AHRs** |
| ***Sociodemographics*** |  |  |  |  |  |  |  |  |
| Age in years  69 – 74 (Ref)  75 – 79  80 – 84  85 + | 1.00  1.10  1.79 ***  1.29 | 1.00  1.04  1.68 ***  1.10 | 1.00  1.02  1.64 ***  1.07 | 1.00  1.04  1.67 ***  1.09 | 1.00  1.04  1.75 ***  1.41 | 1.00  1.02  1.70 ***  1.27 | 1.00  1.00  1.63 **  1.20 | 1.00  1.01  1.68 ***  1.25 |
| Marital Status  Widowed  Divorced/Separated  Never Married  Married (ref) | 1.31 *  0.85  2.16 ***  1.00 | 1.12  0.74  1.70 *  1.00 | 1.11  0.73  1.69 *  1.00 | 1.11  0.74  1.69 *  1.00 | 1.31 *  0.79  2.43 ***  1.00 | 1.12  0.67  1.90 **  1.00 | 1.11  0.68  1.87 *  1.00 | 1.12  0.67  1.88 *  1.00 |
| ***Socioeconomics*** |  |  |  |  |  |  |  |  |
| Education  Grade School  High School (Ref)  College | 1.02  1.00  0.87 | 0.68 **  1.00  1.01 | 0.68 **  1.00  1.01 | 0.68 **  1.00  1.01 | 1.07  1.00  0.87 | 0.73 *  1.00  0.98 | 0.73 *  1.00  0.99 | 0.72 *  1.00  0.98 |
| ***Residence Characteristics*** |  |  |  |  |  |  |  |  |
| Region of the US  Northeast  North Central  West  South (Ref) | 1.10  0.91  0.76  1.00 | 0.93  0.86  0.80  1.00 | 0.92  0.85  0.81  1.00 | 0.92  0.86  0.80  1.00 | NA | NA | NA | NA |
| Type of Residence  Multiple Story Dwelling  Mobile Home  Single Story Non-mobile (Ref) | 1.41 **  1.06  1.00 | 1.42 **  1.09  1.00 | 1.42 **  1.05  1.00 | 1.42 **  1.08  1.00 | 1.42 **  1.14  1.00 | 1.40 **  1.18  1.00 | 1.40 **  1.14  1.00 | 1.40 **  1.18  1.00 |

Table 6. Continued.

|  | **All Persons**  **(N=5,511)^b^** | | | | **Persons Without Baseline Self-Reported Stroke (N=4,987)^c^** | | | |
| --- | --- | --- | --- | --- | --- | --- | --- | --- |
| **Risk Factors** | **Crude HRs** | **Static AHRs** | **Dynamic AHRs** | **Dynamic AHRs** | **Crude HRs** | **Static AHRs** | **Dynamic AHRs** | **Dynamic AHRs** |
| ***Health Behaviors*** |  |  |  |  |  |  |  |  |
| Body Mass  Obese  Overweight  Normal (ref)  Underweight | 1.45 **  0.93  1.00  0.65 | 1.28  0.92  1.00  0.58 | 1.28  0.92  1.00  0.56 | 1.28  0.92  1.00  0.57 | 1.62 **  0.96  1.00  0.72 | 1.54 **  0.98  1.00  0.68 | 1.52 **  0.97  1.00  0.65 | 1.53 **  0.97  1.00  0.67 |
| ***Disease History*** |  |  |  |  |  |  |  |  |
| Arthritis | NA | NA | NA | NA | 0.90 | 0.71 * | 0.70 * | 0.70 * |
| Diabetes | 2.29 *** | 2.01 *** | 1.92 *** | 1.99 *** | 2.32 *** | 2.08 *** | 1.96 *** | 2.04 *** |
| Hypertension | 1.57 *** | 1.38 ** | 1.34 ** | 1.37 ** | 1.51 *** | 1.38 ** | 1.35 ** | 1.37 ** |
| Stroke | 2.14 *** | 1.81 *** | 1.73 *** | 1.79 *** | NA | NA | NA | NA |
| Health Shock (7 day) | 3.43 *** | -- | -- | 2.90 *** | 3.86 *** | -- | -- | 3.37 *** |
| Health Shock (90 day) | 3.18 *** | -- | 2.78 *** | -- | 3.30 *** | -- | 2.98 *** | -- |
| Diff. Picking up a Dime | 1.84 *** | 1.45 * | 1.41 * | 1.44 * | 1.60 * | 1.56 * | 1.53 * | 1.55 * |
| ***Cognitive Status*** |  |  |  |  |  |  |  |  |
| Low ½ Imm. Word Rec.  Refused to Answer Imm. Rec. | 1.63 ***  1.63 | 1.30  0.69 | 1.29  0.71 | 1.29  0.70 | 1.53 ***  1.78 | 1.35 *  1.37 | 1.33 *  1.35 | 1.34 *  1.36 |
| Low ½ Del. Word Rec.  Refused to Answer Del. Rec. | 1.59 ***  2.10 ** | 1.20  2.05 | 1.18  1.96 | 1.19  2.03 | NA | NA | NA | NA |
| TICS-7 Score  Good Cognition | 0.63 *** | 0.74 * | 0.75 * | 0.74 * | 0.62 *** | 0.66 ** | 0.67 * | 0.66 ** |

^a^High Specificity Algorithm: Admission diagnosis of intracerebral hemorrhage, occlusion and stenosis of precerebral arteries, or occlusion of cerebral arteries.

^b^Among the 5,511 AHEAD self-respondents (at baseline), 374 (6.8%) experienced one or more strokes post-baseline and prior to any managed care enrollment.

^c^Among the 4,987 AHEAD self-respondents (at baseline) who did not self-report pre-baseline strokes, 323 (6.5%) experienced one or more strokes post-baseline and prior to any managed care enrollment.

Note: Ref = reference group, NA = not applicable (i.e., risk factor did not make it into this final model).

*p<.05; **p<.01; ***p<.001
